# Changing practice: assessing attitudes toward a NICE-informed collaborative treatment pathway for bipolar disorder

**DOI:** 10.1192/bjo.2021.22

**Published:** 2021-03-08

**Authors:** Adele Louise Elliott, Stuart Watson, Guy Dodgson, Esther Cohen-Tovée, Jonathan Ling

**Affiliations:** Translational and Clinical Research Institute, Newcastle University, UK; Translational and Clinical Research Institute, Newcastle University, UK; and North Locality, Central Business Unit, Cumbria, Northumberland, Tyne and Wear NHS Foundation Trust, UK; Translational and Clinical Research Institute, Newcastle University, UK; and North Locality, Central Business Unit, Cumbria, Northumberland, Tyne and Wear NHS Foundation Trust, UK; North Locality, Central Business Unit, Cumbria, Northumberland, Tyne and Wear NHS Foundation Trust, UK; Faculty of Health Sciences and Wellbeing, University of Sunderland, UK

**Keywords:** Bipolar affective disorders, qualitative research, e-pathway, care pathway, change management

## Abstract

**Background:**

Bipolar disorder is a chronic mental health condition, which can result in functional impairment despite medication. A large evidence base supports use of psychological therapies and structured care in the treatment of mood disorders, but these are rarely implemented. e-Pathways are digital structures that inform and record patient progress through a healthcare system, although these have not yet been used for bipolar disorder.

**Aims:**

To assess the perceived benefits and costs associated with implementing a collaborative NICE-informed e-pathway for bipolar disorder.

**Method:**

Healthcare professionals and people with bipolar disorder attended a workshop to share feedback on e-pathways. Data were collected through questionnaires (*n* = 26) and transcription of a focus group, analysed qualitatively by a framework analysis.

**Results:**

Patients and healthcare professionals welcomed the development of an e-pathway for bipolar disorder. There were five elements to the framework: quality and delivery of care, patient–clinician collaboration, flexibility and adaptability, impact on staff and impact on healthcare services.

**Conclusions:**

Identification of benefits and costs ensures that future development of e-pathways addresses concerns of healthcare professionals and people with bipolar disorder, which would be essential for successful implementation. Recommendations for this development include making e-pathways less complicated for patients, ensuring sufficient training and ensuring clinicians do not feel their skills become invalidated. Limitations of the study, and directions for future research, are discussed.

## Bipolar affective disorder

Bipolar affective disorder has a lifetime prevalence of between 1 and 2.5% of the population.^1,2^ It is diagnosed on the basis of current or prior manic or hypomanic episode(s);^3^ however, low mood that is frequently persistent^4^ and can meet criteria for a depressive episode is more common.^5,6^ The disorder is further characterised by functional impairment^7^ and increased mortality rates, including from suicide.^8^ The estimated cost to the UK is £5.2 billion annually, with direct National Health Service (NHS) costs of £342 million.^9,10^ Biological, psychological and social treatment paradigms are used,^11^ and treatment pathways differ between patients in depressed versus manic episodes, and in patients who are between episodes.^12^ Pharmacological interventions reduce relapse and treat episodes, if used appropriately^12^ Similarly, psychological interventions specifically developed for adults with bipolar disorder improve symptoms and prevent relapse and admission to hospital.^13,14^ These interventions include enhanced relapse prevention, group or individual psychoeducation,^15–17^ cognitive–behavioural therapy and family-focussed therapy.^18^ Group psychoeducation arguably has the strongest evidence base. Research over the past 15 years has concentrated on resource light strategies, with groups integrated within existing treatment pathways (e.g.^19,20^). However, these evidence-based treatments are not widely available within the NHS.

## Care pathways

Care pathways represent a formalisation of the process that underlies care and usually incorporate one or more protocols and guidelines, provide a record of care and a variance record to show where deviations from the planned pathway have occurred.^21^ Computerisation, thereby creating e-pathways, should allow pathways to be integrated with guideline-based decision support, the electronic health record and the clinical workflow.^22^ The entry point to an e-pathway is through an initial care plan. The patient engages in the development of a formulation and the only outcome measure is CollaboRATE, a rating scale for patients that measures how involved they feel in their care. The e-pathways would be accessible to patients, both in the session and accessible through the Cumbria, Northumberland, Tyne and Wear NHS Foundation Trust website, therefore encouraging individuals to be aware of, and request, evidence-based care. The treatment strategies suggested by the e-pathway would be delivered as part of a collaborative care plan. e-Pathways therefore have the potential to improve access to, and use of, the current evidence base and guidelines, to embed values of co-production by building a shared understanding of the difficulties faced by people with bipolar disorder, and to allow formal evaluation of deviations. e-Pathways thus enable identification of resource gaps, training needs and guideline weaknesses. Our NHS mental health trust has an expressed commitment to develop a series of e-pathways that incorporate clinical algorithms. e-Pathways require collaboration between our NHS trust and the electronic patient record provider, and is still under development for bipolar disorder. A pilot showed that the original proposed software package was too time-consuming for clinicians to use. The focus is now shifting to examine options to develop the e-pathway, using the existing capabilities of the electronic patient record supported by a bespoke trust-led software package.

## Algorithm based care

**Fig. 1:**
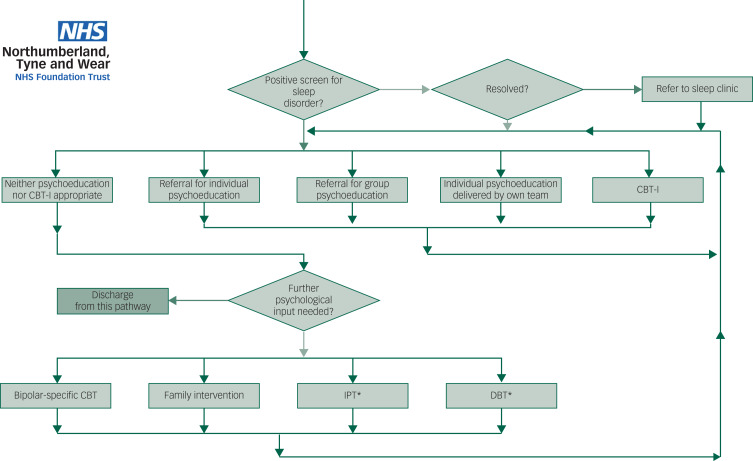
Psychological pathway for out-of-episode bipolar disorder. Note: Entry onto the pathway is determined by a healthcare practitioner that the patient has bipolar disorder and is currently not in episode. Green and red arrows demarcate ‘yes’ and ‘no’, respectively. A diamond box indicates a decision, and rectangular boxes indicate an action. Behind each of these boxes is the necessary information to make a collaborative decision or action, for example: behind ‘Positive screen for sleep disorder?’ there is a rationale for screening for sleep disorders, and a description and screening tools for sleep apnoea and restless leg syndrome. If patients screen positive, the algorithm takes them to the ‘Resolved?’ decision box, where initial advice is given to address the sleep disorder. If this fails to resolve the situation, the algorithm takes the patient and healthcare practitioner to ‘Refer to sleep clinic’, in which information is provided to inform the decision to refer to local clinic, and if appropriate, a referral form. The algorithm next presents a choice of five options, and the information behind the boxes allows the healthcare practitioner to action the choice, or to flag if the resource is not available, e.g. group psychoeducation. The individual can follow the flow chart, e.g. starting with CBT-I and progressing with some individual psychoeducation delivered by the care coordinator, until the individual has confidence to sign up to group psychoeducation. Once this is complete, the patient and healthcare practitioner may feel that psychoeducation or sleep-work is not appropriate. If ‘Further psychological input needed?’ is answered ‘no’, the patient is discharged from the psychological pathway, and continues on the biological and social pathways. An asterisk indicates that it is outside the scope of the National Institute for Health and Care Excellence guidelines. CBT-I, cognitive–behavioural therapy for insomnia; DBT, dialectical behaviour therapy; IPT, interpersonal therapy.

Algorithm-based care refers to the use of clinical algorithms either to aid diagnosis or treatment. Clinical algorithms often comprise step-by-step instructions, often presented in flow-chart form, to guide the clinician.^23^ Studies of unipolar depression have revealed that the use of algorithms markedly improves outcome.^24,25^ In bipolar disorder, the Texas Medication Algorithm Project^26^ has revealed feasibility and efficacy in a public health setting. This has been replicated in a Brazilian study.^27^ Our model incorporates the concurrent use of three algorithms, biological, social and psychological, with different algorithms for patients who are currently in a depressive episode; in a manic, hypomanic or mixed episode; and out of episode (yielding a total of nine algorithms). The algorithms are being produced iteratively, and are based on National Institute for Health and Care Excellence (NICE) guidelines, with support as needed from other clinical guidelines – notably that produced by the British Association of Psychopharmacology^12^ and by the extant evidence base. An example algorithm is shown in Fig. 1.

## Practice change

The movement from the existing structure of care to an alternative in which treatment decisions are made within a framework provided by algorithms, which, in turn, are incorporated into an e-pathway, represents a significant change in practice within our large organisation. Implementation of such a change requires careful consideration.^28^ In healthcare, it has been argued that allowing staff to define problems and formulate solutions facilitates even those change processes that are initiated by managers.^29^ Here, we therefore sought to explore the views of healthcare professionals and people with bipolar disorder; specifically looking at the benefits and costs that are relevant to the development and implementation of an e-pathway for bipolar disorder, to steer and facilitate this change.

## Method

### Study design and setting

Data collection took place in a single session, a day-long workshop organised by the e-pathways team, to present the bipolar disorder e-pathway for discussion.

### Recruitment and participation

Various methods were used to invite healthcare professionals and people with bipolar disorder to the workshop. The workshop was advertised twice via the Trust's weekly bulletin, which is sent to all employees of the Trust. Individuals who had shown earlier interest in the event from a previous bulletin were also directly invited, and invitations were cascaded within the trust, such as via the allied Health Professional Senior Leadership Team. Finally, an open invitation was extended to a bipolar disorder patient/carer group, and other individuals who had previously shown an interest in e-pathways. A combination of open invites and targeted recruitment ensured that feedback from the workshops spanned a variety of occupations and provided a range of perspectives. In total, 28 people attended the workshop. Members of the e-pathway team were also in attendance. Not all attendees provided their job roles within the NHS Trust, although occupations of those in attendance included clinical psychologists, peer-support workers, student nurses, consultants and community psychiatric nurses. Two employees of the NHS Trust in attendance also indicated they had a diagnosis of bipolar disorder, allowing them to provide further insight from the perspectives of healthcare professional and patient. Finally, one person from the bipolar disorder support group was in attendance, as was a carer for a family member with bipolar disorder. In total, three participants had bipolar disorder.

### Format of the workshop

The day included presentations from the bipolar e-pathways team, including presentation of the algorithms that were on display throughout the day, a presentation from a person with bipolar disorder within the Trust, and two focus groups, one of which gave feedback on the pathway during a semi-structured group discussion that incorporated open-ended questions, with prompting for further elaboration. The second focus group explored the training implications of the e-pathway; however, as this was not a focus of the present study, no data were recorded from this group, although all participants from this second focus group completed the questionnaire at the end of the workshop. Attendees had free choice of breakout group. Two of the participants with bipolar disorder attended the focus group that was transcribed.

### Data collection

#### Transcription of focus group

At the start of the discussion, those within the focus group were made aware that the session was to be transcribed. Before beginning, participants were reassured that their responses would remain anonymous. Audio recording was not possible because of logistical constraints. As such, the researcher transcribed the discussion by hand. As much detail was gathered as possible, and where individual fragments were missed, a summary of the speaker's point was instead made. The handwritten transcription was later transferred to computer and checked for accuracy by the researcher.

#### Questionnaire

A questionnaire was designed before the workshop, to explore views about the development of an e-pathway for bipolar disorder. All respondents were made aware of the purpose of the questionnaire. The questionnaire (see Table 1) was distributed and completed at the end of the day. Following the session, responses were compiled into a document for analysis

**Table 1:** Bipolar e-pathway questionnaire

	Question
1	How do you think an e-pathway for people with bipolar disorder will affect the quality of care you/the Trust deliver/receive?
2	Would you welcome the development of such a pathway? What do you see as the advantages?
3	Do you have any concerns about the pathway? Are there potential negative consequences?
4	In what way do you think support, guidance and/or training could be developed to improve the quality of care you deliver/receive?
5	Do you have any further comments about the Bipolar Pathway?
6	Would you be interested in helping to develop the pathway? If yes, please give your contact details below

Data were analysed by framework analysis,^30^ to systematically establish relationships within the data to answer relevant questions through the generation of a framework. This method of analysis, developed for applied policy research, is becoming increasingly used within healthcare and medical research.^31^ It allows the flexibility to examine ideas that arise from the data during analysis.^32^ Themes can be described as concepts that aim to describe the data.^31^ We followed the five stages to framework analysis: familiarisation, identification of a thematic framework, indexing, charting, and mapping and interpretation.

### Ethical approval

As a service evaluation, this study did not require consent to be granted from Newcastle University nor NHS Research Ethical Committees. However, all procedures contributing to this work comply with the ethical standards of the relevant national and institutional committees on human experimentation and with the Helsinki Declaration of 1975, as revised in 2008. Participants acknowledged their consent to discuss the topic before participating in group discussions.

## Results

In total, 26 questionnaires were submitted at the end of the workshop. All respondents indicated that they welcomed the development of the e-pathway for bipolar disorder. Through analysis of the questionnaires and focus group, we developed an analytic framework that comprised five key concepts: quality and delivery of care, patient–clinician collaboration, flexibility and adaptability, impact on staff and impact on healthcare services.

### Quality and delivery of care

Participants were asked how they felt the quality of care, and care delivery, would be affected as a result of implementation of e-pathways. Overall, healthcare professionals and patients felt that the implementation of an e-pathway for bipolar disorder would improve care. A key theme was that the consistency of care would improve, and that there would be a sense of equality of care. Another benefit for the implementation of e-pathways was that it was felt that clinicians would be more likely to deliver care concordant with NICE guidelines, which would be associated with improved outcomes for patients.

‘I'd hope it will create a more consistent baseline to good practice in offering evidence-based interventions whilst understanding the subtleties of why sometimes we alter our treatments’ (questionnaire, no role given).

Furthermore, a theme arose from the discussion that having standardised care and the ability to record treatment progress would allow clinicians who regularly deviate from NICE guidelines, without sufficient justification, to be ‘flagged’. This could assist with highlighting areas where care is consistently substandard.

A further concept that arose was that e-pathways would be expected to facilitate the development of evidence-based and value-based treatment strategies. One healthcare professional indicated:

‘I think it has enormous potential in improving the quality of care of service users and helping staff in deciding/delivering evidence-based practice’ (questionnaire, consultant clinical psychologist).

Furthermore, another respondent felt that the e-pathway would help to:

‘[i]mprove the consistency of approach – aligned to the evidence base of what works [and] support recording of decision making about treatment and variance from NICE recommendations’ (questionnaire, psychologist).

Overall, following analysis, the idea that the quality and delivery of care would improve was widespread; however, care must be taken to ensure equality of availability of interventions.

### Patient–clinician collaboration

The collaborative relationship between clinician and patient as a result of e-pathways, including a more holistic approach to care, increased co-production, and increased patient engagement also emerged from the data. A presumed benefit of e-pathways would be the ability to include the patient in the decision process of treatment, as both clinician and patient could view the algorithm within e-pathways, and discuss options relating to each stage or intervention. Most questionnaire respondents raised the idea of increased collaboration as a result of e-pathways, with many also indicating they believed it would be a more holistic model of care.

‘It should improve quality outcomes and service user experience by ensuring informed decisions about elements of care packages, ensuring consideration to be given to a holistic bio-psycho-social approach […] better engagement with interventions and service due to collaborative transparent approach’ (questionnaire, no role given).

However, a potential barrier arose in the physical appearance of the algorithm:

‘Showing [a] patient the visual form would be overwhelming. I feel overwhelmed looking at the flow chart. It could be easier to follow or more friendly looking or I would not show it to a patient’ (focus group, psychologist).

Therefore, consideration must be given to the appearance of the decision-tree, or how it could be better presented to patients to prevent this being a barrier.

The final element of collaboration related to family members or carers, with one healthcare professional indicating e-pathways would be:

‘[An] extra resource to utilise when working with clients and their families’ (questionnaire, no role given).

e-Pathways were perceived as providing an effective way to maintain collaboration between the patient and those involved in their care, including carers, family members and clinicians.

### Flexibility and adaptability

Flexibility and adaptability refer to how e-pathways for bipolar disorder could change to provide optimal care. In relation to concerns regarding implementation of e-pathways, one respondent indicated:

‘Possibility of being a little rigid but [a] clinician can utilise [their] own clinical decision making to justify care and treatment going forward in care plans, progress notes, etc.’ (questionnaire, community psychiatric nurse).

Participants emphasised the importance of ensuring that clinicians were aware of the scope to use their own clinical judgement to deviate from the treatment algorithm, with justification and to adapt treatment to suit specific groups and to be flexible to accommodate management of other co-morbidities. One concern that arose from several healthcare professionals related to individuals with co-morbidities and adapting e-pathways to suit other groups, such as adolescents or the elderly:

‘Need to ensure co-morbidities are understood and that people don't forget about problems that don't fit under this diagnosis’ (questionnaire, no role given).

Healthcare professionals suggested that broadening the e-pathway process to support both pre-engagement and recovery would be important in improving care for patients with bipolar disorder. For example, pre-engagement could include enabling the patient to be able to access resources relating to the algorithms and/or interventions before commencing treatment.

‘If public-facing it could introduce the patient early for [their] own research or mood diaries’ (focus group, consultant).

Similarly, healthcare professionals believed it would be beneficial if e-pathways could be adapted to support both post-engagement recovery.

### Impact on staff

Respondents reported that they felt that the implementation of e-pathways would have an impact on staff such as clinicians and mental health nurses, and that clinicians would benefit from the more structured guidance and clearer expectations, which may increase clinician confidence.

‘I think a downstream advantage may be that clinicians come to supervision with clearer expectations and questions e.g. ‘We got stuck doing X, how can I approach this with the client?’, which would drive the quality of care’ (questionnaire, psychologist).

‘[The] principle of an e-pathway which will guide clinicians is excellent, having information and interventions/guides will enhance confidence’ (questionnaire, consultant clinical psychologist).

However, sufficient guidance was felt to be needed to ensure that individuals were aware of their job role expectation with regard to delivery of e-pathways:

‘There must be greater clarity re job role expectations – who is expected to deliver what and how this fits within [the] broader job role’ (questionnaire, psychologist).

One benefit that was identified was the idea of an aspirational pathway, that e-pathways provided the standard of care that should be aspired to, which could give staff a clearer sense of purpose and boost morale. However, this could present a possible barrier: some clinicians stated that they may feel frustration if they are unable to deliver the recommended standard of care such as through a lack of resources. One respondent indicated that this could be ‘demoralising’ (focus group, practitioner adolescent services). Another indicated:

‘[The] clinician may become overwhelmed if there are no staff resources to develop care or do not have training to implement certain groups or one to one session’ (questionnaire, no role given).

This sense of an impact on staff was mirrored by a potential impact on patients: disappointment if a recommended intervention was not available in all areas. This was thought likely to be especially difficult for individuals in rural areas, or those with limited mobility that are unable to travel:

‘[The] risk of it being a “postcode lottery” – shows what should be offered but if not available in that service [due to lack of funding in a particular location]; it would be frustrating for the service user’ (questionnaire, no role given).

A further cost stemmed from concerns regarding increased pressure and demands on staff:

‘Support to engage with the pathway, staff feeling overwhelmed and overloaded and pressured to discharge’ (questionnaire, consultant clinical psychologist).

Although some indicated they felt implementation of e-pathways could be time-saving and reduce their workload, others indicated the pressures staff already faced could present an issue in terms of uptake. A further factor that could affect staff is the idea of ‘process-driven care’. Several healthcare professionals voiced concerns that e-pathways would become a ‘tick-box exercise’ (questionnaire, perinatal mental health team).

‘We need to ensure we are using it with purpose, not because we have to’ (questionnaire, no role given).

One issue was that the implementation of e-pathways would become a method of monitoring staff performance as opposed to a tool to provide guidance and resources. One respondent on the questionnaire indicated that, despite the stated purpose of e-pathways to provide support for and not to assess clinicians, they were concerned that it may ‘become part of a performance framework’ (questionnaire, no role given).

Also, a key issue to implementation was the concern that it ‘takes the art out of nursing’ (questionnaire, no role given) or ‘[I] worry that it might unwittingly invalidate care working skills’ (questionnaire, psychologist); namely, that it may lead to a loss of instinct and clinical judgement.

### Impact on healthcare services

This concept relates to how implementation of e-pathways would affect healthcare services, with concerns relating to cost, training and resources.

‘My underlying concern is how it would be implemented, resources, staff, training […] and being put into practice’ (focus group, practitioner adolescent services).

One key benefit raised was that implementation of a system such as e-pathways would facilitate the auditing process:

‘Agree it may help to highlight gaps in resources and help us think about how to tackle this’ (questionnaire, no role given).

For example, if the treatment algorithm regularly recommends an intervention that is not available in one locality, it is easier to identify where care or resources are falling short.

A barrier with a potential to affect the implementation of e-pathways was training, with many healthcare professionals indicating the necessity of sufficient training to ensure correct implementation and use of e-pathways:

‘Need to establish [the] training needs of staff of different professions and peer support workers and experts by experience. Some of this will be awareness and care skills, some related to specific interventions’ (questionnaire, no role given).

A specific concern related to training around interventions, as many believed there were currently insufficient practitioners trained in the interventions that would be recommended within the treatment algorithm. This relates to similar concerns regarding availability of resources. A further concern related to insufficient supervision being in place to support training and implementation.

## Discussion

Overall, healthcare professionals and patients welcomed the development of the e-pathway for bipolar disorder, and helped to define several benefits supporting the implementation of e-pathways, including improvements in the quality and consistency of care, increase in good practice and NICE-concordant care, clearer guidelines resulting in increased satisfaction for clinicians, a more collaborative approach to care and ability to monitor care and resources. If e-pathways are to be successful, the benefits identified by patients must remain central to development and implementation. However, it is arguably of at least equal importance to establish the barriers that could hinder implementation in order for necessary solutions to be developed. Costs and possible recommendations are discussed below.

In keeping with previous research,^33^ healthcare professionals feared loss or invalidation of care skills, clinical instinct and confidence in unsupported clinical decision-making by trainees. The potential liability consequent on deviations from the algorithm was also a concern. This highlights the importance for the pathway team of adequately communicating that the standard of care recommended in e-pathways is aspirational, and that healthcare professionals should feel able to use clinical judgement and deviate from the algorithm. This further emphasises the importance of collaborative algorithm development.

The algorithm was seen as ‘overwhelming’ for healthcare practitioners and patients because of its apparent complexity. A further concern was that irrelevant aspects of the treatment algorithm would still be visible for some patients, such as the inclusion of medications that could be contraindicated. One individual suggested that a more user-friendly version, such as a simplified paper handout, could be developed. A further suggestion was that a ‘step’ within the algorithm could change colour to indicate when it is completed, to make the visual aspect of the flow, charts more intuitive and easier to follow from a patient perspective. These recommendations need to be considered in the e-pathway design and a combined approach may be needed in which the healthcare practitioner and patient are able to see the whole algorithm to give a sense of the treatment journey and future options with a more focused view of the immediately relevant treatment decisions. Healthcare practitioners felt that effective training and resources would be central to successful implementation for e-pathways. This replicated experience elsewhere,^34^ and is a timely reminder that sufficient resources need to be allocated to training

It was notable that although the bipolar e-pathway team saw the identification of gaps between best practice and actual practice as an important function of the e-pathway – a way of identifying gaps in training and provision, and of informing service development – these gaps appeared to raise anxieties in the workshop attendees. This will need careful consideration during the process of e-pathway development.

This project had several limitations. First, as this was a valuable opportunity to have access to a task-specific group of healthcare professionals and people with bipolar disorder, the research had to be as pragmatic as possible. As such, only one researcher undertook data collection and because of logistical constraints, and data from only the most relevant focus group were transcribed. Further detail, which may have helped to answer the research questions, may have therefore been missed. Because of the lack of transcription, it may have been beneficial to validate the derived transcript and arising themes with participants; however, this was not possible within the current study. Additionally, one researcher carried out the qualitative analysis, which may have made it more susceptible to subjectivity. The researcher also developed the analytical framework, and this was discussed and refined with other members of the team.^35^ A further limitation is that few people with bipolar disorder responded to the invitation to the workshop, and therefore feedback was primarily from healthcare professionals. Although clinicians will use e-pathways in day-to-day practice, implementation will affect patients through the care and interventions they receive. As such, it is vital to gain an understanding of the views of patients in the development and implementation process. Therefore, this process would have benefitted from further contributions from people with bipolar disorder. One patient in attendance agreed that they would take the concept of e-pathways to their bipolar support group, with feedback shared in a less formal setting, and their input will help to shape the development of the pathway.

This research assessed attitudes toward a treatment pathway that is yet to be implemented. Although it is essential to ascertain stakeholders’ views throughout development, it is also important to assess the system once in practice. Future research should examine the use of e-pathways once implemented. This could either be a quantitative assessment of the magnitude of change in care, using outcome measures as described in studies above, such as ratings on self-report measures and rates of hospital admission. If the e-pathway functions correctly, it would be predicted that patients with bipolar disorder will have lower rates of readmission and relapse. A qualitative methodology, as used here, could examine healthcare professionals and patients’ views (e.g. at the onset of implementation and 1 year later), to determine whether the perceived benefits of e-pathways are realised in practice, and if the costs identified in the current research are sufficiently overcome.

In conclusion, the provision of e-pathway-supported, algorithm-informed care has huge potential to inform service development, identify training needs, enhance collaborative clinical decision-making, streamline processes and improve quality of care. There are considerable hurdles to overcome before the development and subsequent delivery can occur, but an optimised e-pathway has the potential to improve outcomes and equity for patients with bipolar disorder.

## Data Availability

Data available on request due to privacy/ethical restrictions.
